# Holliday Junction Thermodynamics and Structure: Coarse-Grained Simulations and Experiments

**DOI:** 10.1038/srep22863

**Published:** 2016-03-14

**Authors:** Wujie Wang, Laura M. Nocka, Brianne Z. Wiemann, Daniel M. Hinckley, Ishita Mukerji, Francis W. Starr

**Affiliations:** 1 Department of Physics, Wesleyan University, Middletown, Connecticut 06459, USA; 2 Department of Molecular Biology and Biochemistry, Wesleyan University, Middletown, Connecticut 06459, USA; 3 Molecular Biophysics Program, Wesleyan University, Middletown, Connecticut 06459, USA; 4 Department of Chemical and Biological Engineering, University of Wisconsin-Madison, Madison, Wisconsin 53706, USA

## Abstract

Holliday junctions play a central role in genetic recombination, DNA repair and other cellular processes. We combine simulations and experiments to evaluate the ability of the 3SPN.2 model, a coarse-grained representation designed to mimic B-DNA, to predict the properties of DNA Holliday junctions. The model reproduces many experimentally determined aspects of junction structure and stability, including the temperature dependence of melting on salt concentration, the bias between open and stacked conformations, the relative populations of conformers at high salt concentration, and the inter-duplex angle (IDA) between arms. We also obtain a close correspondence between the junction structure evaluated by all-atom and coarse-grained simulations. We predict that, for salt concentrations at physiological and higher levels, the populations of the stacked conformers are independent of salt concentration, and directly observe proposed tetrahedral intermediate sub-states implicated in conformational transitions. Our findings demonstrate that the 3SPN.2 model captures junction properties that are inaccessible to all-atom studies, opening the possibility to simulate complex aspects of junction behavior.

DNA four-way or Holliday junctions are central intermediates in the cellular processes of recombination, integration and DNA repair. Such structures have also been implicated in fork reversal during replication and regulation of gene expression^1^. These structures typically form from inverted base repeats of 6 base pairs or longer^2^. Investigations of the human genome have revealed that short inverted repeats often occur in regions of high chromosomal instability and breakage, leading to cancer and other diseases^2^. These non-B-DNA structures are well recognized by different classes of proteins, including junction-resolving enzymes, which are critical for maintaining genome stability, architectural proteins and other regulatory proteins^1^.

DNA junctions can exist in two broad classes of conformations, open and stacked (Fig. 1)^3^. The open conformation of the junction is usually described with the four arms at approximately 90° to each other with no central base stacking. Atomic force microscopy studies suggest that these 4-fold symmetric structures have a square, planar configuration^4^,^5^. In this conformation the junction is capable of branch migration between homologous duplexes, an essential step for recombination and repair processes. The stacked conformation, which is induced through the binding of metal ions, leads to the formation of two quasi-continuous helices with pairwise stacking of the helical arms (Fig. 1b,c). In this form, the rate of branch migration is significantly slowed, presumably as a consequence of reduced sampling of the open form^6^,^7^,^8^.

Crystallographic studies of relatively short homologous junctions have provided much information regarding the details of junction molecular structure. In the absence of proteins, junctions have crystallized in the stacked form, stabilized by backbone-base and backbone-ion interactions^9^,^10^,^11^. In addition, crystal structures of protein-junction complexes have allowed visualization of other configurations, such as the open form and other hybrid structures^12^,^13^,^14^. Spectroscopic studies of immobile junctions prepared from non-homologous sequences suggest that, in the stacked form, the arms adopt an anti-parallel orientation, which does not require a crossing of the strands in the central region^3^. In this orientation, the junction can adopt two forms, iso-I and iso-II, which are stereochemically equivalent but use different arms for the stacking partners. Experimental studies, particularly single-molecule fluorescence measurements, have revealed that, in solution, the junctions can dynamically interconvert between these different structures^15^,^16^,^17^. The bias towards one stacked structure (iso-I) over the other (iso-II) is largely dependent on the base sequence in the central region of the junction^18^,^19^. The propensity of the junctions to adopt a folded or stacked conformation, depending on the salt concentration of the solutions, has been exploited in the development of DNA nanostructures (see ref. 20 and references therein). Elucidating the factors that stabilize junction structure is critical to the use of junctions as components in the building of nanostructures, and to our overall understanding of how junctions behave in the cell. Development of a robust computational model that can predict structural, energetic, and dynamical properties of junctions is an important tool to make progress towards these goals.

The modeling of DNA by all-atom, classical force fields (including solvent), such as the CHARMM^21^ and AMBER^22^ models, has proven immensely useful to describe many DNA properties, including molecular conformations and the structure of protein-DNA complexes. Several computational methods have been applied to the study of junction structure and dynamics, providing some insights into the overall behavior^23^,^24^,^25^,^26^,^27^. For example, Westhof and co-workers used Brownian dynamics to identify ion-binding sites in the junction^27^. More recently, all-atom simulations have been used to examine junction conformation, dynamics and the interaction with proteins – effectively reproducing some of the key structural features of such complexes^24^,^25^. However, the computational complexity of these highly-detailed models makes it challenging to address many important structural and energetic features, such as conformational transformations and DNA hybridization. These challenges are illustrated by a recent study of junction isomerization dynamics, where it was demonstrated that conformational sampling can be non-ergodic over time scales of up to one minute^26^. Accordingly, a number of efforts have been made to develop coarse-grained representations of DNA that reduce the number of degrees of freedom and simplify or eliminate calculation of the solvent^28^,^29^,^30^,^31^,^32^,^33^,^34^,^35^,^36^,^37^,^38^. These models are able to capture length and time scales that are intractable from atomistic models. Thus, in this study we combine simulations and experiments to investigate the ability of a coarse-grained model to predict junction thermodynamics and conformational populations.

The 3-site per nucleotide (3SPN) model has been demonstrated to be particularly successful in quantitatively reproducing the experimentally measured thermodynamic and structural properties of B-DNA, including melting temperature, persistence length, and salt effects^32^,^34^,^35^. The model is parameterized using a top-down experimentally informed approach, rather than a bottom-up coarse-graining of all-atom representations. The validity of the model to describe more complex, non B-DNA structures, such as the Holliday junction, has not been tested to this point. Thus, we examine the ability of the 3SPN.2 DNA model^32^ to describe thermodynamic and structural properties of the well-studied J3 Holliday junction, originally developed by Lilley and co-workers^40^,^42^. Here, the junction is truncated so that each arm has 17 bases, and we refer to it as J34. Importantly, conformer populations, overall dynamics and junction angles for J3 configurations have all been experimentally determined^15^,^16^,^43^,^44^,^45^, providing a good basis for comparison with computation and our ongoing experiments. Additionally, we provide experiments for the salt concentration dependence of melting thermodynamics. As discussed above, the junction occurs in open and stacked forms, and for the specific case of J3, single molecule and bulk Förster resonance energy transfer (FRET) studies have shown that the iso-II conformer is preferred in solution at a roughly 3:1 ratio^15^,^44^.

In this study, we show that the 3SPN.2 model reproduces our experimentally measured concentration dependence of junction melting, the bias between open and stacked conformations, as well as the relative population of stacked isomers at high salt concentration. Our simulations provide new results regarding the concentration dependence of the relative populations of open and stacked conformations as a function of salt concentration; specifically, we find that the population of the iso-I and iso-II forms is nearly independent of salt concentration at concentrations where the stacked (rather than open) conformation dominates. Furthermore, these simulations also reveal that considerable conformational heterogeneity exists within the broad categories used to classify junction structure. In particular, we find that open tetrahedral conformations, rather than planar open states, play an important role in conformational transitions between iso-I and iso-II conformations at high salt concentration, in agreement with the proposed intermediate states based on all-atom simulations^23^,^24^. We also compare the junction structure of iso-II in the 3SPN.2 model, as measured by the inter-duplex angle (IDA), to both FRET experiments and new all-atom calculations using the AMBER force field^22^. Overall, we find that the 3SPN.2 model is highly successful in reproducing many J3 junction properties, and at the same time we identify some of the limitations of this coarse-grained representation.

## Results

### Junction Melting

As a first step to assess the applicability of the 3SPN.2 model to predict bulk thermodynamic properties of the Holliday junction, we evaluate the junction melting properties from simulations of the 3SPN.2 model and compare it with our absorption experiments. We simulate the junction melting via replica-exchange molecular dynamics (REMD, see methods) to improve the sampling of partially melted states. Accurate sampling of the relative fraction of intact, melted, and partially melted junction configurations is not presently possible using an all-atom representation.

Figure 2 shows the single-strand fraction *α* as a function of *T* over a range of salt concentrations for both the 3SPN.2 model and absorbance measurements. See methods for the definition of *α* in simulations. The similarity between the simulations and experiments is striking. Both simulations and experiments show that the melting temperature increases with increasing salt concentration, and that the transition becomes increasingly sharp, or “cooperative”. The data can be well described by the law of mass action, or van’t Hoff relation^46^,


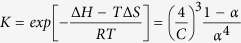


where *K* is the equilibrium constant for junction melting, Δ*H* and Δ*S* are the enthalpy and entropy difference between the melted and intact junction, which are assumed to be constant over this temperature range, and *C* is the total strand concentration (the concentration of each strand is *C*/4). Note that ΔS controls the sharpness of the melting transition. We will discuss the behavior of Δ*H* and Δ*S* below, which are determined from fitting data in the range 0.1<α<0.9.

The melting temperature *T*_*M*_ is a useful thermodynamic metric of junction stability, and is commonly defined as the temperature at which *α* = 1/2. Figure 3 shows the experimental and simulated *T*_*M*_ values as a function of salt concentration. As anticipated from the melting curves, the simulations closely mirror the experimental dependence of *T*_*M*_. The quantitative similarity is also remarkable. The simulation results are consistently shifted to higher *T* by 5 to 8 K, a difference of less than 3%. For both experiments and simulations, the melting temperature is nearly independent of salt concentration for [Na^+^] ≥ 200 mM. This independence is related to the screening of electrostatic interactions at high salt concentrations; accordingly, Fig. 3 also shows the Debye screening length used in the 3SPN.2 model for the Debye-Hückel approximation of the electrostatic interactions. The screening length is <6 Å at high salt concentration, making electrostatic repulsion insignificant. The correspondence between *T*_*M*_ values of the experiment and simulation validates the approximate treatment of the electrostatics in the 3SPN.2 model. Given that the 3SPN.2 model was only parameterized to mimic the melting of duplex B-DNA, the junction melting data suggest that the 3SPN.2 model is transferable to the thermodynamics of more complex DNA structures. At the same time, this finding is not entirely unanticipated, since the melting of each arm should be very similar to that of ordinary duplex DNA.

The fit of the data using the van’t Hoff relation (equation 1) provides an estimate of the enthalpy and entropy of melting. The values of Δ*H* and Δ*S* from this fit are of the correct order of magnitude that would be expected based on the thermodynamic parameters determined by SantaLucia and co-workers^47^,^48^. The inset of Fig. 4 shows that the simulations modestly overestimate Δ*H* and Δ*S* values, relative to the experiments, and that Δ*H* varies nearly linearly with Δ*S*. This linear relation is sometimes referred to as a “compensation” relation. In fact, the van’t Hoff equation implies a specific relationship between Δ*H* and Δ*S*


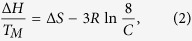


which follows from eq. 1 by setting *α *= 1/2 at *T*_*M*_. Figure 4 demonstrates consistency with this relationship, and clarifies that linear compensation between Δ*H* and Δ*S* is only approximate; if *T*_*M*_ values varied more significantly with concentration, the deviations from linearity between Δ*H* and Δ*S* would be more apparent, while eq. 2 should remain valid. More important from a practical standpoint is that these results demonstrate the effectiveness of the 3SPN.2 model in reproducing energetic and thermodynamic characteristics of the junctions that are in close agreement with the experimental data.

### Conformational Abundances and Structure

**Figure 1:**
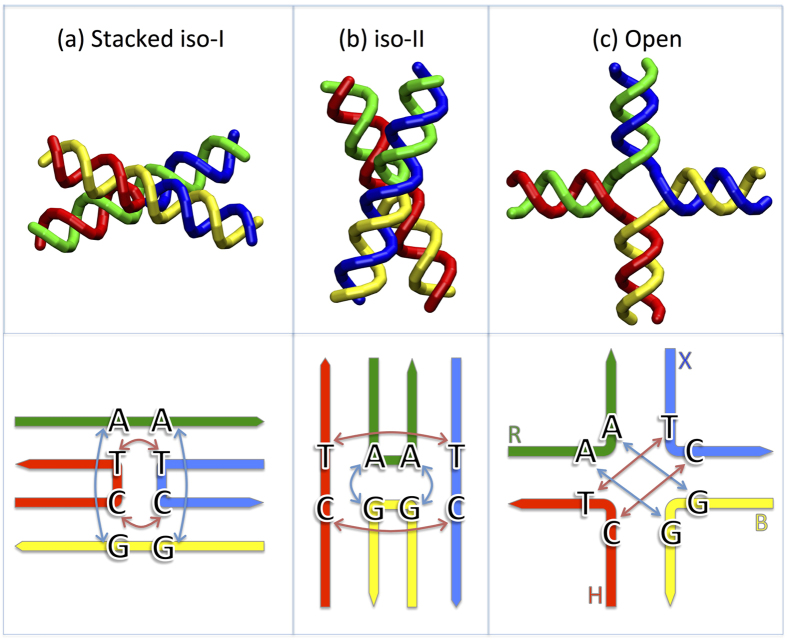
Idealized schematic of the junction conformations. The stacked (**a**) iso-I and (**b**) iso-II conformations predominate at modest and high salt concentrations. (**c**) The open conformation is mainly observed at low salt concentration, and also potentially acts as an intermediary of conformational changes between iso-I and II. The colors denote the strands of the J3 junction, labeled X, R, B, and H, following the convention of Lilley and co-workers^39^,^40^,^41^. See methods for the complete sequence. In the lower panel, the arrows highlight the inter-base distances for non-complementary bases of distinct strands at the junction center. In iso-I, the TT and CC separations are small (red arrows), while the AG distances are large (blue arrows); the opposite occurs for iso-II. The distances are essentially identical in the open conformation. We take advantage of these differences to distinguish conformations.

**Figure 2:**
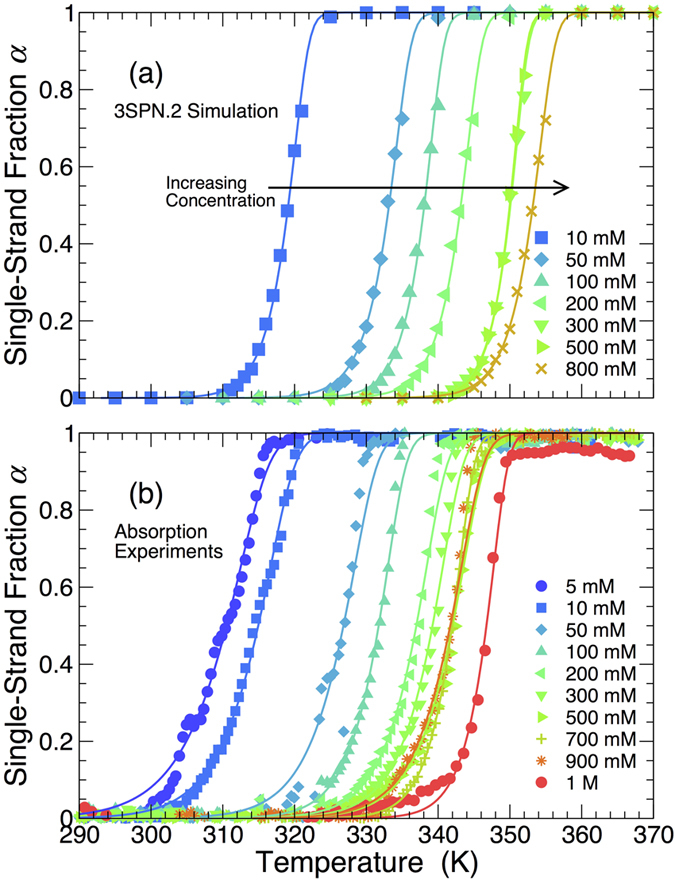
Melting properties of the J34 junction. Comparison of the single-strand fraction *α* from (**a**) simulations and (**b**) experiments. We fit the data using the van’t Hoff equation (equation 1), indicated by the solid lines. Following convention, we define *T*_*M*_ as the temperature at which *α *= 0.5.

**Figure 3:**
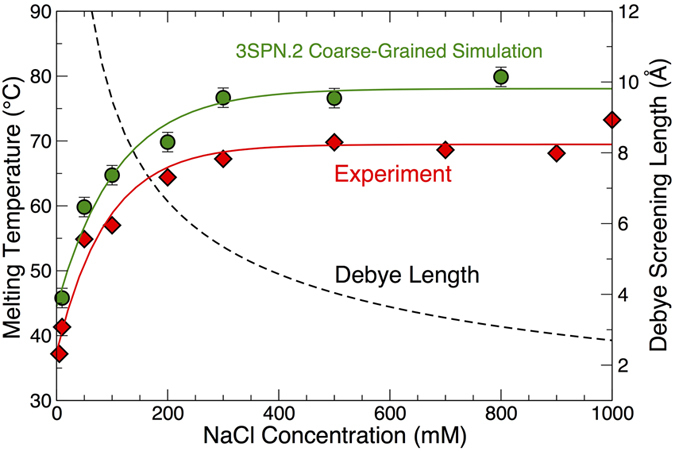
Salt concentration dependence of the junction melting temperature *T*_*M*_. At all concentrations, *T*_*M*_ differs by <3% between 3SPN.2 simulations (green circles) and absorption experiments (red diamonds). The lines are only intended as a guide to the eye. The figure also shows the Debye screening length used by the 3SPN.2 model in the Debye-Hückel approximation to the electrostatic interactions. The plateau of the melting temperature coincides with strong screening of electrostatic interactions.

**Figure 4:**
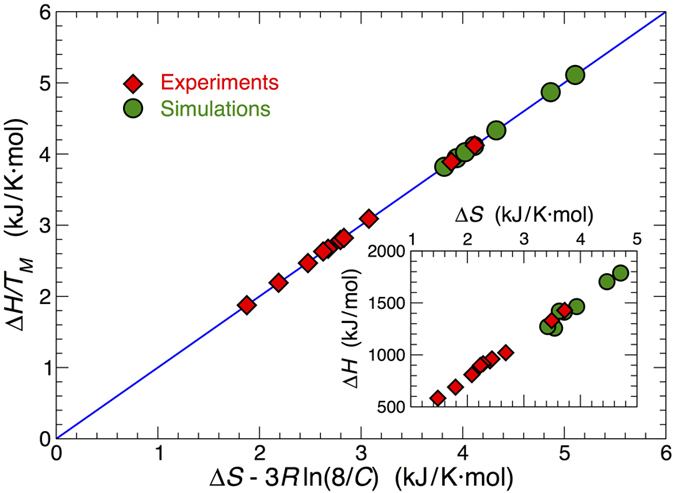
Thermodynamics of junction melting. The entropy Δ*S* and enthalpy Δ*H* of melting obtained by fitting the experiments (red diamonds) and simulations (green circles) to the van’t Hoff equation (equation 1). The inset shows an approximate linear entropy-enthalpy compensation relation. Both the experimental and the simulated data show better agreement with the van’t Hoff equation evaluated at *T*_*M*_ (eq. 2, blue line).

To further probe the ability of the 3SPN.2 model to predict experimental junction properties, we examine the relative population of junction conformations. Experimentally, it is known that the open planar form is predominant at low salt concentration, and, at higher salt concentration, junctions adopt stacked conformations (Fig. 1). At a relatively high concentration [Mg^2+^]* *= 50 mM and *T *= 298 K, experiments indicate a 23/77% relative abundance of stacked isoforms I/II, respectively^15^. Here, we use the 3SPN.2 model to examine a broad range of salt concentrations at *T *= 300 K to validate the model and provide a prediction for the overall salt concentration dependence of junction populations. Note that we examine the effect of Na^+^, rather than Mg^2+^, which yields similar results^16^,^45^.

As Thirumalai and coworkers emphasized^26^, the correspondence between a time average and the ensemble average (ergodicity) for four-way junctions can be broken over rather large observational time scales, due to the rate of conversion between junction conformations. Thus, to estimate the relative populations of the stacked conformers, we need a significant ensemble of simulations. Accordingly, for each salt concentration, we carry out 100 independent simulations, each starting from the open configuration, since this conformation rapidly and randomly converts to either iso-I or iso-II. Each simulation is run for 2 μs, yielding 200 μs total of trajectory data for each salt concentration, split among 100 independent samples. This provides an initial estimate for the relative population of each conformation. Based on this estimate, we run 100 further simulations using a ratio of open, iso-I or iso-II initial configurations that conform to the preliminary population estimate in order to see if our results are sensitive to starting from the open state. We find that the population estimates remain stable within our statistical uncertainty, so that we do not find any significant effects due to starting in the open state. Our results are affected if we use either all iso-I or all iso-II initial configurations, due to the relatively slow inter-conversion of these states.

To evaluate the junction populations, we need to identify a metric that accurately distinguishes among the three primary junction conformations. Experimentally, FRET studies commonly use the distance between the ends of select arms (which defines the inter-duplex angle) to distinguish conformational states. We initially examined inter-duplex angle, but found (as is discussed later) that the IDA fluctuates substantially in each isoform, making it difficult to unambiguously distinguish conformations. Instead, we found that the inter-base separations near the heart of the junction provide a more reliable indicator of junction conformation. These distances should also be accessible experimentally using a pair of fluorescent nucleotide base analogs, such as 6-methyl isoxanthopterin (6-MI) or 2-aminopurine, placed judiciously close to the junction center. The lower panels of Fig. 1 show the eight bases at the core of the junction in the iso-I and iso-II conformers, as well as the open form. In the iso-I conformer, the separation *d*_*TT*_ of the T bases (on strands X and H) and the separation *d*_*CC*_ of the C bases (also on strands X and H) are much smaller than the separations *d*_*AG*_ of the A and G bases (on the B and R strands); in the iso-II conformer, the reverse stacking leads to the opposite behavior of the relative distances. In the open form, all of these distances are similar, but larger than the distances of the stacked iso-I and II conformers.

**Figure 5:**
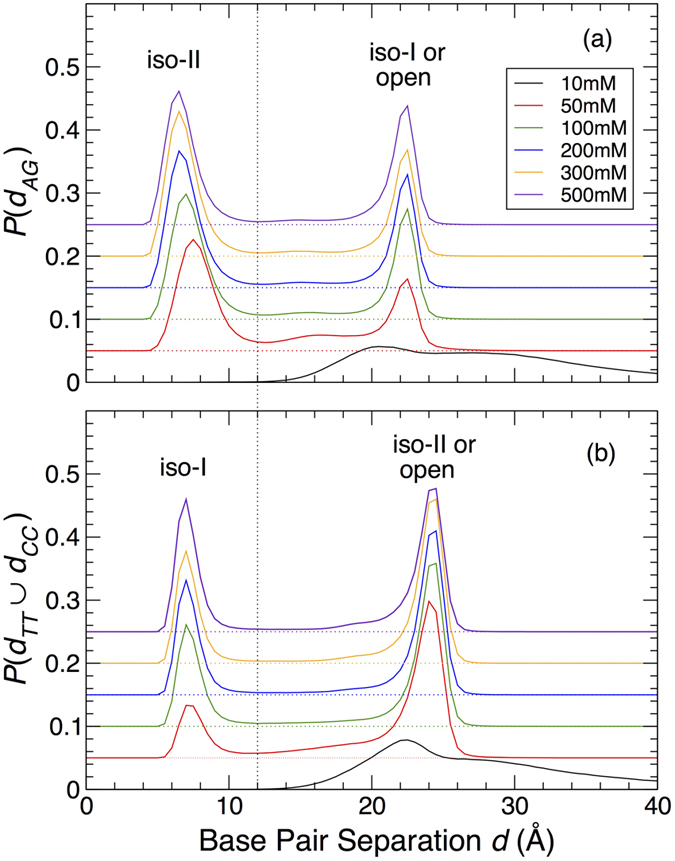
Criterion for distinguishing junction conformations. Distribution of inter-base separation at the middle of the junction for (**a**) the AG bases, where a small separation identifies the iso-II conformer, and (**b**) TT or CC pairs, where a small separation identifies the iso-I conformer. The longer distance peak at low salt concentration is due to open conformations; at higher salt, it arises primarily from the complementary stacked form. The vertical dotted line indicates the cutoff criterion we use to subsequently identify conformational states of individual configurations.

Therefore, we should be able to distinguish between iso-I and II simply based on these inter-base separations. To confirm this, in Fig. 5 we plot the normalized frequency distribution of inter-base distances *P*(*d*_*TT*_ ∪ *d*_*CC*_) and *P*(*d*_*AG*_), which demonstrates the presence of two distinct peaks for all concentrations ≥50 mM. For *P*(*d*_*AG*_) (Fig. 5a), the peak at small separation indicates the iso-II conformer states, and the peak at larger separation is due to either iso-I or open conformations. For *P*(*d*_*TT*_ ∪ *d*_*CC*_) (Fig. 5b), the peak at small distances indicates iso-I states, and the peak at larger distances are either iso-II or open conformations. We adopt a cut-off of 12 Å separation, the approximate location of the minimum of the distributions, to distinguish junctions as either the stacked iso-I or iso-II conformer. Those configurations that are identified as neither iso-I nor II are classified as open conformers. Consistent with the expectation that the open conformation dominates at low salt concentration, neither distribution has a peak for small separation, and so nearly all configurations at low salt are identified with the open conformation.

**Figure 6:**
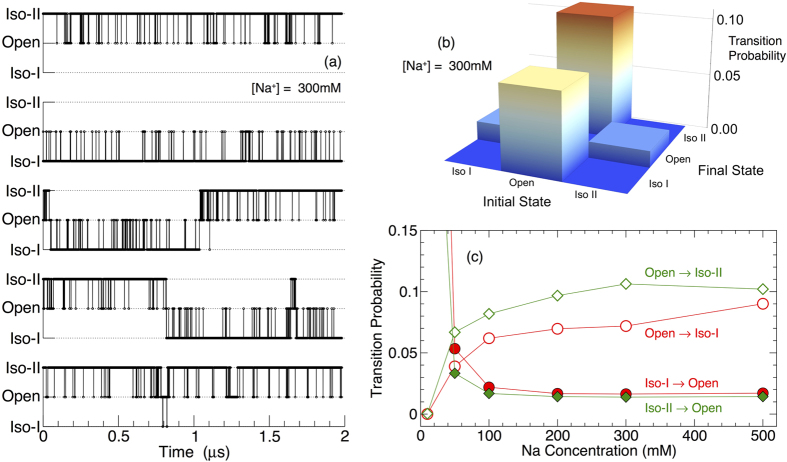
Molecule-to-molecule variations in conformational sampling and conformational transition probabilities. (**a**) Example time series of junction conformations for five of the 100 ensemble members at [Na^+^]* *= 300 mM. The open isoform is short lived, and acts a transition state between iso-I and iso-II. (**b**) Matrix of the transition probabilities from a given starting state to final state at [Na^+^]* *= 300 mM. The transition probabilities to the same state (diagonal elements) are not shown, since the tendency to remain in the current state dominates the scale of other transition probabilities^49^. Note that the transition probabilities for iso I→II (and vice-versa) are nearly zero. (**c**) Salt concentration dependence of the four key transition probabilities.

**Figure 7:**
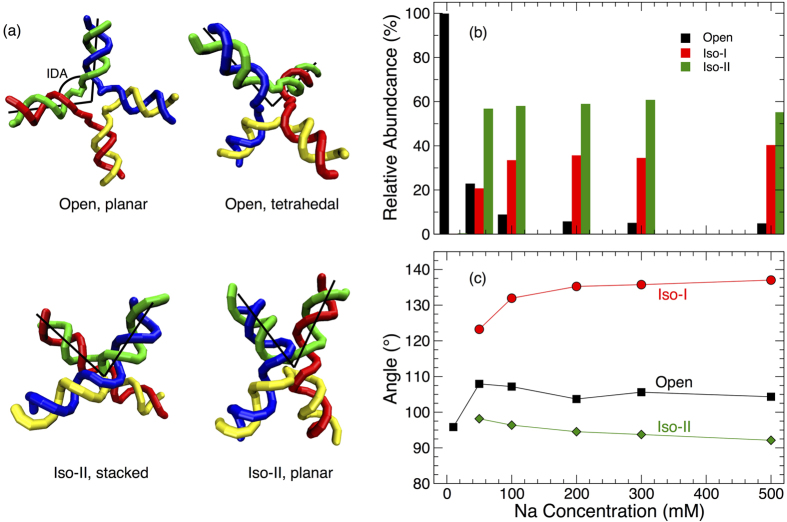
Junction conformations, abundance, and structure. (**a**) Representative conformations observed in our simulations; only the DNA backbone is shown, for simplicity. (**b**) The relative abundance of the primary isoforms as a function of salt concentration. Isoform identities are defined by the base separations at the junction interior, as described in the text. (**c**) Inter-duplex angle (IDA) for each conformation. Note that the open planar conformation is only predominant at low salt. At higher salt, the small fraction of open conformations sampled adopt (on average) a tetrahedral conformation.

Using these criteria to distinguish conformers, we identify individual configurations with one of the three isomers, and show representative time series of junction conformations for five simulations at [Na^+^]* *= 300 mM in Fig. 6a. Figure 7a shows representative configurations of these conformers. The time series data qualitatively show that the open conformation is extremely short lived, and acts as a transition state between the long-lived iso-I and iso-II conformers. This has previously been inferred from single molecule experiments^8^,^15^,^16^,^50^, and in this study, we explicitly observe and confirm the transition mechanism between equilibrium conformer states. We quantitatively demonstrate the role of the open conformation as an intermediate by evaluating the nine transition probabilities (including transitions to the same conformation) among the three conformers. Figure 6b shows that the transition probabilities for Iso I→II and II→I are vanishingly small on the scale of these plots (zero, within the uncertainly of our calculation). Consequently, the only path between the stacked conformers is through the open conformer, establishing it as the transition state. Figure 6c shows how the transition probabilities between the stacked and open conformations vary with salt concentration, with transitions to the open state dominating at low salt concentrations. The five time series in Fig. 6a also demonstrate that the time average of the data varies from sample-to-sample. Clearly 2 μs is inadequate for an ergodic sampling of conformational abundances, and necessitates our approach of using an ensemble of simulations. This approach is possible for the coarse-grained model, but is beyond present computational resources for an all-atom model. This “broken ergodicity” is similar to the experimental finding that conformational sampling is non-ergodic on large times scales^26^. However, we should be careful to point out that the broken ergodicity here is entirely due to the stochastic nature of conformational sampling, while in the experiments of ref. 26, variations in the binding of ions to individual junctions also play a role.

Based on the time series data for junction conformation, we directly evaluate the fraction of configurations in the open, iso-I or iso-II conformations as a function of salt concentration (Fig. 7b). As we expect, for low salt concentrations ([Na^+^]* *= 10 mM), we observe essentially only the open planar state with square geometry. At all higher salt concentrations, the stacked conformers are preferred, with an average of ≈58% in iso-II, and 36% in iso-I (for [Na^+^] ≥200 mM). Our simulations predict that, except for rather low salt concentrations, the relative fraction of conformers does not depend significantly on salt. The dominance of stacked conformations at high salt concentration can be expected due to the strong screening of electrostatic interactions, similar to the salt concentration dependence of the melting temperature. However, this screening does not account for the bias toward the stacked iso-II conformation. The difference in isomer population must arise due to base sequence effects near the junction core, as observed experimentally^16^,^17^. The fact that the model reproduces the experimentally known bias toward iso-II indicates the degree of success of the coarse-grained model in capturing sequence dependent structure. We also find a small fraction, 6%, of junction configurations in the open state at high salt. As discussed above, these conformations are short lived, and facilitate transformations between the stacked conformations. Compared to the experiments of Ha and coworkers^15^ at relatively high salt concentrations, the 3SPN.2 model under predicts the bias toward iso-II by about 15%. The difference may be due in part to the difference in experimental and computational criteria used to define isoforms; specifically, we examine the base separations at the junction interior, while the FRET experiments use labels which are sensitive to the separation of the ends of the R and X arms of the junction (effectively the IDA). For H-like conformations with an open junction center (observed experimentally in the presence of endonucleases^12^), these alternate approaches would systematically differ in their classification: (i) based on junction ends, H conformations would be classified as iso-I or II; (ii) conversely, based on separation at the junction core, H-conformations would be classified as open, so that our criteria will always yield a smaller stacked fraction.

Since our criteria allow us to categorize the conformations of individual junction configurations, we can also directly evaluate the mean structure of each of these conformations. We characterize the junction structure by the inter-duplex angle (IDA), which we define by the angle formed by the XR and RH arms with the junction vertex^51^. Accordingly, the IDA for iso-II should be substantially smaller than that of iso-I. Figure 7c confirms this expectation, with the IDA of iso-II approaching 90° at high salt concentration, and the IDA of iso-I near 140°. A value of 90° for iso-II is large compared with experimental estimates, which are on the order of 40–60° based on equilibrium FRET measurements^41^,^44^,^45^, a point we shall return to. We should also be clear that 90° for iso-II does not imply a planar configuration, which is visually apparent in the representative configurations shown in Fig. 7a; owing to the three-dimensional structure, even a stacked structure can adopt an angle close to 90°. At the lowest salt concentrations, we cannot estimate the angle for stacked conformations, since they are essentially not sampled. At [Na^+^]* *= 10 mM, we find the IDA of the open junction is 95°, consistent with a nearly planar junction, which is also visually apparent in Fig. 7a. At higher salt concentrations, the small fraction of open configurations adopts a somewhat larger angle of ≈105°. This larger angle is consistent with a nearly tetrahedral configuration (Fig. 7a), which should perhaps be considered distinct from the planar open configuration at low salt concentration. Thus, our results suggest that transition states between stacked conformers are predominantly open tetrahedral conformations, rather than planar. Such tetrahedral type intermediates have been observed previously in simulations^23^. Indeed, the possibility of a multitude of junction conformations has been inferred from analysis of single molecule data^26^.

### Junction Structure Comparisons

**Figure 8:**
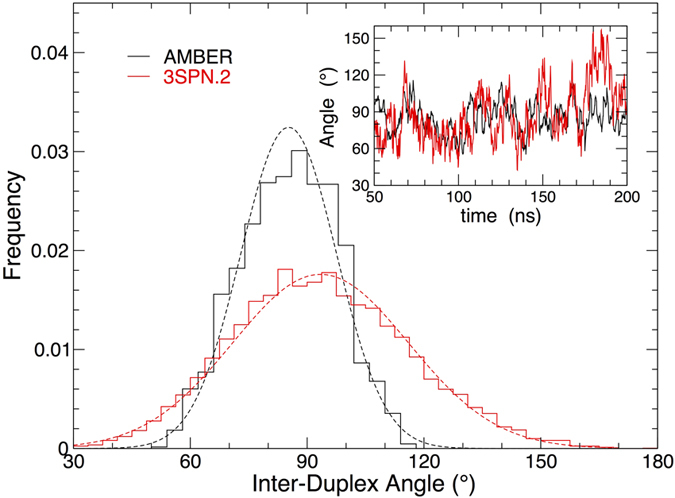
Junction structure comparison between all-atom and coarse-grained models. The IDA for the all-atom AMBER model (black) and 3SPN.2 coarse-grained model (red) at T* *= 283 K and [Na^+^]* *= 200 mM. The main panel shows the distribution of sampled IDA values; solid lines are the calculated frequency, and dotted lines are a normal distribution with the same mean and standard deviation as the data. The inset shows the original time series for each model, from which the distributions are determined. Note that for the 3SPN.2 model, we have 2 μs of data, not all of which are shown in the inset.

As a final point of comparison, we consider how the IDA evaluated from the 3SPN.2 model compares with experimental measurements, and that estimated from our all-atom AMBER simulation. FRET measurements have estimated the IDA of iso-II is 43 ± 8° at T = 283 K and salt concentration [Na^+^] = 200 mM^45^. Accordingly, we simulate both the AMBER and 3SPN.2 models under matching conditions, starting from an initial iso-II configuration with an IDA of 43° (see Fig. 1b). For both the 3SPN.2 and AMBER models, the junction opens to a larger IDA, and settles to a steady value after ≈50 ns (inset Fig. 8). The main panel of Fig. 8 shows the IDA values sampled by each model in the steady state. The 3SPN.2 model exhibits broader fluctuations of the IDA than the AMBER model, although the mean IDA is similar in the two models. Quantitatively, for AMBER, the mean IDA is 85.3° with standard deviation 12.3°; for 3SPN.2, the mean IDA is 95.7° with standard deviation 24.1°.

The simulations both show a substantially larger IDA than anticipated from the FRET measurements and crystallographic studies^45^,^52^. This may suggest that neither model provides an accurate estimate of the IDA, or that the approximations that must be made to estimate IDA from the FRET data underestimate the IDA. Certainly, in the bulk FRET experiments the presence of the external dyes, their associated linkers and their relative orientation can introduce considerable error into the distance determination. The differences between the current IDA determinations and those measured by crystallography could perhaps arise from some constraints of the crystal lattice. In comparing the all-atom and coarse-grained models, the near doubling of the standard deviation clearly shows that the junction arms are more flexible in the coarse-grained model. This may be due in part to the absence of explicit solvent, since the solvating water in the all-atom representation necessarily hinders the flexibility of the junction arms.

## Discussion

We have examined the thermodynamic and structural properties of a four-way junction and demonstrated the applicability of the coarse-grained 3SPN.2 model to mimic an array of experimental junction properties. The 3SPN.2 model nearly quantitatively reproduces the temperature dependence of junction melting on salt concentration. Given that the model was designed to reproduce the melting properties of B-DNA, this success is not entirely unanticipated, as the arms of the junction closely resemble standard DNA helices. However, the structure near the junction core includes unstacked bases and a distorted backbone, which is significantly different from B-DNA, and so the success in describing properties that depend on junction core structure is noteworthy. As in experiments, the 3SPN.2 model folds the junction and exhibits a bias towards the stacked iso-II conformation with increasing salt. Furthermore, the simulations allow us to predict that the relative populations of isoforms is nearly constant for [Na^+^]≥100 mM. In doing so, we introduced a new metric to identify junction conformations based on the base separations at the interior of the junction rather than the distance between the arms. This approach should be feasible to examine experimentally using fluorescent nucleotide base analogs, such as 6-MI and 2-AP; we are currently developing such experiments.

Owing to the computational expediency of the 3SPN.2 model, we gain new insight regarding the transitions between the stacked conformations. As a result, we are able to confirm the proposal that the open conformation serves as an intermediate state for transformations between the iso-I and iso-II conformers^15^,^16^. Furthermore, we also demonstrate that the previously proposed tetrahedral variant of the open form^23^, rather than the planar form, is the average transitional conformation. Significantly, we also observe a planar version of the iso-I and II conformers, which is consistent with previous FRET experiments where a kinked X-structure was found to best describe the data^53^. The observation of such structures emphasizes that the family of open and stacked conformations consists of a multitude of sub-states, which may be important for function^26^.

One of the main advantages of these coarse-grained models is that they allow for the exploration of questions that simply are not feasible using all-atom models with current computational resources. For example, the ability to examine a significant ensemble of simulations allows us to obtain reliable population estimates of conformers. Similarly, we are able to explicitly evaluate junction stability and our estimates of *T*_*M*_ are in good accord with experiments. These favorable comparisons to experiments, as well as the similar IDA of the 3SPN.2 and the all-atom AMBER models, indicate that the 3SPN.2 model can provide predictive results for four-way junction properties. Thus, the model offers a promising platform to examine the molecular mechanics of the junction melting process, as well as the mechanics of the conformational transitions and branch migration. By the same logic, coarse-grained representations of RNA hold the promise to help unlock the complexities of RNA folding, and there is already substantial progress in this direction^54^,^55^,^56^,^57^,^58^,^59^,^60^,^61^.

The 3SPN.2 model naturally has limitations, demonstrated by the significant difference in the mean IDA relative to experiments, as well as the enhanced IDA fluctuations relative to the AMBER calculations. The origin of the discrepancy in the mean IDA is not readily apparent, since the AMBER simulations reveal nearly the same mean IDA as the 3SPN.2 model. The enhanced fluctuations in the 3SPN.2 model are likely related to the implicit nature of ions. Specifically, experiments^42^,^62^ and all-atom simulations^23^,^24^ demonstrate that ions tend to concentrate near the junction core, significantly impeding core rearrangements^26^. On the other hand, the absence of explicit ions has a beneficial effect, as it facilitates conformational transitions. As a result, the dynamics of conformational sampling are enhanced relative to experiments and all-atom studies. Thus, the enhanced fluctuations can be viewed as both a drawback and a benefit. We are currently initiating simulations using an explicit ion version of the 3SPN.2 model^63^ to better explore the effect of ions on dynamics. In the implicit case, predictions for junction dynamical properties in the 3SPN.2 model will need to be scaled to provide quantitative predictions for experiments. Such enhanced dynamics are not uncommon in coarse-grained models, and have been discussed for the coarse-grained MARTINI model for membranes^64^. Naturally, the coarse-graining of the 3SPN.2 model precludes examination of many internal atomic details of DNA structure. However, the direct mapping from all-atom coordinates to the sites of the 3SPN.2 model suggests that the 3SPN.2 model could potentially be coupled to all-atom calculations, serving as a tool to enhance large-scale conformational sampling.

The degree of success in describing junction properties is highly encouraging. Consequently, the model holds promise to provide insights into a variety of complex DNA structures. Ultimately, such coarse-grained representations need to be combined with coarse-grained models for proteins and other biomolecules, to enable the examination of more complex bimolecular interactions and mechanisms that cannot be readily studied by all-atoms models. Indeed, a coarse-grained representation of DNA has been developed for the MARTINI model very recently^38^, which already has models for membrane and protein simulation. These efforts promise to open a vast array of new comparisons between experiments and molecular modeling.

## Methods

### 3SPN.2 coarse-grained modeling

The 3SPN model, introduced in 2007^34^, uses three coarse-grained sites for each nucleotide: one each for the sugar, phosphate group, and base. This representation allows for resolution of major and minor grooves using isotropic potentials. Further refinements of the model (3SPN.1^35^ and 3SPN.2^32^) improved the description of ionic strength, sequence, concentration, and temperature dependence of duplex formation. The most recent refinement of the model also accurately reproduces the persistence lengths of both single-stranded and duplex DNA, and predicts reaction rate constants that are consistent with experimental values. This model continues to be actively developed and improved upon by the de Pablo research group^65^.

Like atomistic models, the 3SPN.2 model uses a combination of classical bonded and non-bonded interactions between the coarse-grained sites. Bond potentials include a combination of bonding, bending, and diherdral interactions. Non-bonded interactions are carefully parameterized to reproduce experimental properties, and include excluded-volume interactions, intra-strand stacking, inter-strand cross-stacking, and base-pairing interactions. Electrostatic interactions are included between charged phosphate sites, which are screened using the Debye-Hückel approximation to account for solvent effects. The screening length and permittivity are parameterized to account for salt concentration and temperature. There are no explicit solvent or ions sites, although a version of 3SPN that includes ions and solvent has been developed^63^,^66^. For complete details of the 3SPN.2 model, please refer to ref. 32.

To simulate the 3SPN.2 model, we use the LAMMPS molecular dynamics (MD) suite^67^, modified to incorporate the 3SPN.2 potentials using the freely available source^65^. Integration of the equations of motions for the 3SPN.2 model is done via Langevin dynamics, as a way to incorporate random and frictional solvent effects on the evolution of the system. We follow the parameters of Hinckley *et al.*^32^, who use an integration time step of 20 fs. This is roughly one order of magnitude larger than the time step for all-atom simulations. Combined with the dramatically reduced complexity of the DNA representation, these features make microsecond scale simulations of duplex DNA of modest length easily accessible. As a consequence, direct examination of the DNA melting transition is feasible, although ensuring ergodicity of individual simulations can be challenging, given the heterogeneity of conformational sampling among ensembles of single junctions^26^.

**Figure 9:**
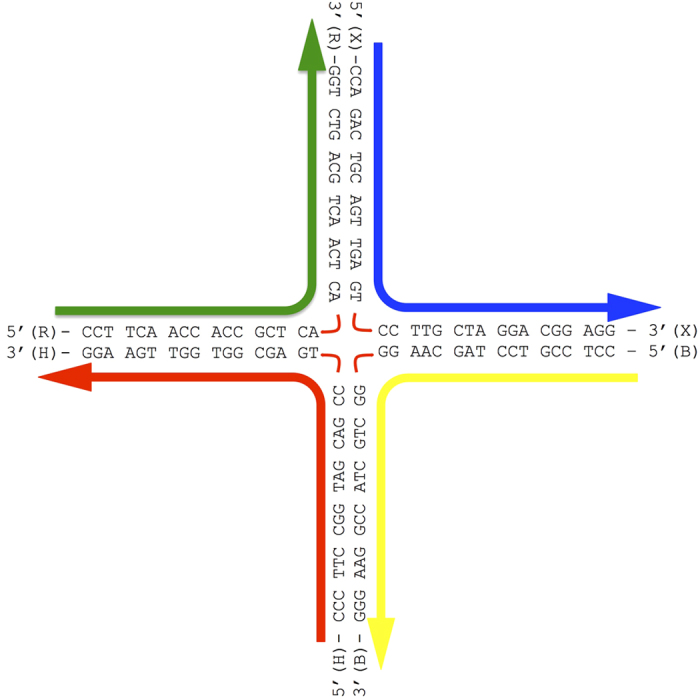
Sequence of the junction J34. Each arm is 17 bp long, a truncated version of the J3 junction.

In the 3SPN representation, the J34 Holliday junction requires only 404 force sites; see Fig. 9 for the complete sequence. Simulations use an initial configuration that is either an idealized open or stacked conformation (Fig. 1); we specify the initial state for specific calculations in the main text. Generally, the open configuration readily transforms to a stacked conformation at high salt concentration for T ≥ 300 K, making the choice of initial configuration unimportant.

Even for this coarse-grained model, accurate estimation of the melting transition is challenging. To improve our sampling of intermediate, partially hybridized states, we take advantage of replica exchange molecular dynamics (REMD)^68^. The REMD method is a hybrid MD/Monte Carlo scheme that allows for the exchange of configurations among separate simulations that are carried out at different temperatures. Provided that the temperature difference between simulations is small enough that their energy distributions significantly overlap, the exchange process can significantly enhance the effectiveness of sampling across the entire temperature range. To wisely select the temperatures for the chain of REMD simulations, we first perform a set of ordinary MD simulations, each 6 μs in duration, covering a broad range of temperature, to crudely estimate the melting location. We use configurations from these initial simulations to seed a chain of 17 to 20 REMD simulations, which are carried out for 6 μs, and we attempt exchanges at 200 ps intervals during the course of the simulation.

Since the absorption experiments provide an estimate of the relative fraction of single strands, we make a similar estimate from our REMD trajectories. To do so, we implement a criterion to define intact and melted junction strands, since configurations can have a broad range in the number of intact base-pair bonds. For complementary base pairs in the junction, we consider a bond intact when the base-pair separation is less than 10 Å; any choice in the range of 7 to 12 Å does not qualitatively affect our findings. We then define a strand as ‘single’ if more than half of the possible base-pair links are not intact^30^; the results for our melting curves are only weakly sensitive to the precise criterion used, over the range from 40 to 60% of unlinked base pairs.

### AMBER all-atom modeling

For the purpose of comparing the inter-duplex angle as estimated from FRET experiments, the 3SPN.2 model, and all-atom DNA models, we have carried out an all-atom simulation of the J34 junction using the AMBER model for DNA at T = 283 K and salt concentration [Na^+^]* *= 200 mM at ambient pressure. Temperature and salt are chosen to match experimental conditions^45^. The junction simulation utilized a CUDA enabled PMEMD version of the AMBER12 simulation suite^69^. We use the parm99 force field with the parmbsc0^70^ and ions08^71^ modifications for DNA and monovalent ions, respectively. We solvate the systems in an octahedral box using the TIP3P model for water molecules^72^, yielding a total of 135,111 atoms (as compared to the 404 sites for the 3SPN.2 representation). Non-bonded van der Waals interactions are cutoff at 9 Å, and we handle long-range electrostatic interactions using the particle mesh Ewald method^73^,^74^. We adjust pressure and temperature using the Berendsen method^75^; we use the SHAKE algorithm for rigid bond constraints^76^ and a simulation time step of 2 fs. At this salt concentration, the iso-II conformer predominates experimentally, and we use this isoform for the initial configuration. To prepare the system, we minimize the energy of a solvated junction, and then rapidly heat to 283 K over 20 ps; we then collect data for a further 200 ns, discarding the first 50 ns of data. Simulations of this time scale are accessible by taking advantage of GPU computing resources.

### Junction preparation and absorption experiments

Oligonucleotides were purchased from Integrated DNA Technologies (Coralville, IA) and gel purified as described in ref. 45. The sequences of the junction strands are shown in Fig. 9. Preparation of junctions follows our published methods^45^. DNA melting experiments were monitored by UV absorption and performed on a Beckman-DU spectrophotometer. Junction concentrations of 150 nM ensured that the sample absorbance remained in the linear range (0.1–1.0) over the course of the melt. All measurements were performed in a 10 mM Tris-HCl, pH 7.6, 0.1 mM EDTA buffer with Na^+^ concentrations as indicated. Absorption of junction samples at 260 nm was monitored from 5 to 95 °C at a resolution of 1°/pt and a ramp rate of 0.5°/min. Samples were saturated with helium before melting and the sample chamber was flushed with nitrogen gas to prevent condensation on the cells. The contribution of the buffer was subtracted from all of the melts prior to analysis. Thermodynamic melting parameters were calculated from the melts using the formalism described in the main text.

## Additional Information

**How to cite this article**: Wang, W. *et al.* Holliday Junction Thermodynamics and Structure: Coarse-Grained Simulations and Experiments. *Sci. Rep.* **6**, 22863; doi: 10.1038/srep22863 (2016).
